# Plant-Derived Organic Acids Are Linked to Arbuscular Mycorrhizal Fungi and *phoD*-Harboring Bacteria Associated with Improved Soil Phosphorus Availability Across Plant Functional Groups in Karst Ecosystems

**DOI:** 10.3390/microorganisms14050952

**Published:** 2026-04-23

**Authors:** Shu Zhang, Fujing Pan, Yueming Liang, Kelin Wang, Zijun Liu, Wei Zhang

**Affiliations:** 1 Guangxi Key Laboratory of Environmental Pollution Control Theory and Technology, College of Environmental and Engineering, Guilin University of Technology, Guilin 541006, China; zhangshu_mail@126.com (S.Z.); panfujing@glut.edu.cn (F.P.);; 2 University Engineering Research Center of Watershed Protection and Green Development of Guangxi, Guilin University of Technology, Guilin 541006, China; 3 Key Laboratory of Carbon Emission and Pollutant Collaborative Control, Education Department of Guangxi Zhuang Autonomous Region, Guilin University of Technology, Guilin 541006, China; 4 Karst Dynamics Laboratory of Ministry of Natural Resources, Institute of Karst Geology, Chinese Academy of Geological Sciences, Guilin 541004, China; 5 Huanjiang Observation and Research Station for Karst Ecosystem, Guangxi Key Laboratory of Karst Ecological Processes and Services, Institute of Subtropical Agriculture, Chinese Academy of Sciences, Changsha 410125, China

**Keywords:** karst ecosystem, available phosphorus, AMF, *phoD*-harboring bacteria, organic acid

## Abstract

Phosphorus (P) limitation is prevalent in terrestrial ecosystems. Plants can improve soil P availability through the exudation of organic acids and symbiotic interactions with microorganisms. However, associations between different plant functional groups and phosphorus cycling in P limited karst ecosystems remain poorly understood. To investigate this, the exudation rates of oxalic, citric and acetic acids from fine roots, the contents of carbon, nitrogen, and P in leaves and fine roots, and the contents of oxalic, citric and acetic acids, total P, available P (AP), and microbial biomass P in rhizosphere soils were measured across different plant functional groups in a karst ecosystem in southwestern China. Additionally, the activities of acid and alkaline phosphatases were also analyzed, as well as the relative abundance, community structure, diversity, and co-occurrence network patterns of arbuscular mycorrhizal fungi (AMF) and alkaline phosphatase-encoding (*phoD*) gene-harboring bacteria. The results showed that both the exudation rates and the contents of organic acids and AP were highest in the tree group, followed by the shrub and grass groups. The AP content of the legume group was significantly higher than that of the non-legume group. The exudation rates of oxalic acid were significantly greater than those of citric and acetic acids. AMF diversities were highest in the shrub and legume groups. The diversities of *phoD-*harboring bacteria decreased from the tree group to the shrub group and then to the grass group, yet there were no significant differences between the legume and non-legume groups. The communities of both AMF and *phoD-*harboring bacteria exhibited significant differences among these plant functional groups. The prevalent genera of *phoD*-harboring bacteria across all groups were *Pseudomonas* and *Halomonas*, with *Halomonas* being particularly prevalent in the legume group. The AMF community was dominated by *Glomus*, which attained its highest relative abundance in the tree and legume groups. Furthermore, the increased exudation rate and content of oxalic acid were associated with higher relative abundances of *Glomus* in AMF and *Pseudomonas* and *Bacillus* among *phoD*-harboring bacteria. Structural Equation Model (SEM) analysis demonstrated that plant-exuded organic acids, especially oxalic acid, were positively associated with P availability indirectly through their linkages with the diversity and abundance of AMF and *phoD*-harboring bacteria. The crucial role of oxalic acid was particularly prominent in the tree and legume groups. Our findings suggest that screening AMF and *phoD*-harboring bacteria with highly efficient P transformation activity and inoculating them into the rhizosphere of plants with high oxalic acid exudation could help improve plant resilience to P limitation and support sustainable restoration in karst ecosystems.

## 1. Introduction

Phosphorus (P) is a limiting element for plant growth [1]. P limitation is widespread in terrestrial ecosystems, particularly in karst ecosystems [2,3]. A study has shown that in karst ecosystems, plants are initially limited by nitrogen (N), then progress to co-limitation by N and P, and ultimately become limited by P with advancing vegetation restoration [4]. This limitation is particularly pronounced in low-latitude regions [5]. This phenomenon is primarily related to the fact that the parent material in karst regions is predominantly carbonate rock [6]. This parent material has a native P content ranging from 0.2 to 0.6 g/kg [7,8], which is lower than that (0.5~1.2 g/kg) of non-karst regions [9,10]. Secondly, P combines with calcium to form inorganic P fractions such as calcium-bound P (Ca_2_-P, Ca_8_-P, Ca_10_-P), iron-bound P (Fe-P), aluminum-bound P (Al-P), and occluded P (O-P) [11]. These fractions constitute 70% to 90% of the total P in these regions [12]. Thirdly, historical over-cultivation has led to soil and water loss through subterranean flows, with an annual available P loss estimated at 2.2~5.5 kg/hm^2^ [13,14]. For example, soil P levels in cultivated or degraded areas are 20~40% lower than those in natural vegetation [15,16]. Therefore, a crucial challenge in karst ecosystems is to supply available P for plants.

Current research indicates that plants have evolved multiple strategies associated with higher P uptake capacity, including plastic regulation of root architecture, exudation of organic acids, establishment of mycorrhizal symbiotic networks, and expression of efficient P transporters [17,18]. Among these, root exudation of organic acids represents a key strategy for plants to overcome P limitation [19], functioning through two primary pathways: direct P solubilization and indirect microbial regulation.

Organic acids can directly dissolve bound P [20]. Low-molecular-weight organic acids from root exudation solubilize P in calcareous soils through Ca chelation and acidification [21]. Chelation occurs when multiple carboxyl groups (-COOH) in organic acids like oxalic acid and citric acid form stable complexes with Ca. For instance, oxalic acid can precipitate calcium oxalic upon excess Ca, releasing P from bound Ca-P complexes [22]. Acidification, conversely, involves organic acids releasing H^+^ through the dissociation of their carboxyl groups, promoting the protonation of PO_4_^3−^ into H_2_PO_4_^−^ [23]. Recent studies further reveal significant variations in the types of organic acids, exudation strategies, and their efficiency in promoting P activation across different ecosystems. In non-karst regions, the exudation of citric acid and malic acid by plants primarily activates Fe-P, Al-P, and organic P [24,25]. However, in karst systems, high calcium concentrations and alkaline conditions drive oxalic acid as the dominant exudation—characterized by low pKa values (pKa_1_ = 1.25, pKa_2_ = 4.14) and strong Ca chelation capacity (logK(CaOx) = 4.6) [26]. This targeted strategy enhances P activation by providing carbon sources and shaping the rhizosphere microbial community, thereby strengthening microbial P solubilization pathways [27]. While oxalic acid is frequently the dominant exudate in calcium-rich karst environments due to its high calcium chelation efficiency, this study specifically included citric and acetic acids to represent a broader spectrum of low-molecular-weight organic acid (LMWOA) configurations. Citric acid, a tricarboxylic acid, provides a contrasting high-affinity chelating potential, whereas acetic acid, a monocarboxylic acid, serves as a simpler carbon source and proton donor, allowing for a comparative analysis of how varying carboxyl group densities influence phosphorus (P) solubilization kinetics across different plant functional groups.

Organic acids can also serve as carbon sources for microorganisms and are linked to the optimization of microbial community structure and function to enhance P availability [28,29]. As carbon sources for soil microorganisms, organic acids are associated with the synergistic P transformation mediated by arbuscular mycorrhizal fungi (AMF) and functional microorganisms [30]. For instance, in regions with high soil calcium carbonate content, such as the Mediterranean, plants like olive trees, through the exudation of malic acid, promote the expansion of arbuscular mycorrhizal fungi (AMF) mycelium networks, thereby broadening P uptake [31,32,33]. Another study indicates that woody plants rely on oxalic acid to synergistically release P from the occluded phosphate (O-P) fraction with AMF [24]. In regions with low soil calcium carbonate content (10~15%), such as southern Spain, plants like olive trees and thyme establish stable symbiotic relationships with AMF through the preferential exudation of malic acid and tartaric acid [34]. In these systems, the AMF mycelium network can contribute 40~60% of total plant P uptake [35]. Furthermore, plants can also cooperate with free-living microbes. *PhoD*-harboring bacteria possess genes encoding key subunits of alkaline phosphatase (ALP). Under low P stress, *phoD* gene expression is upregulated, enhancing ALP exudation to ultimately mineralize organic P and release available P [36]. Furthermore, we prioritized the assessment of *phoD*-harboring bacteria as a primary functional index. Among the various genes encoding alkaline phosphatases (ALP), *phoD* is recognized as one of the most prevalent and environmentally sensitive markers for bacterial organic P mineralization in terrestrial soils. While acid phosphatase (ACP) and other enzymes such as phytase contribute to organic phosphate availability, the neutral-to-alkaline pH levels characteristic of karst lithosols suggest that alkaline-mediated mineralization pathways, specifically those governed by *phoD*-carrying communities, may exert a more significant influence on P cycling during vegetation restoration than their acid-responsive counterparts. Focusing on the *phoD* gene provides a genetic proxy for potential organic P mineralization. While gene abundance does not directly equate to realized enzyme activity, our simultaneous measurement of ALP activity allows for a robust cross-validation of microbial functional capacity in these calcareous soils. In tropical regions, plants synergistically interact with free-living microorganisms via root-derived citric acid to solubilize iron-aluminum-bound P (Fe-P/Al-P) [37] and mineralize organic P [38].

In natural ecosystems, different plant functional groups have evolved diverse organic acid exudation strategies. However, in karst ecosystems, the high-calcium, high-pH soil environment strongly influences organic acid types, making oxalic acid—due to its strong calcium chelation capacity and low synthesis cost —the dominant exudation. While the general pathway linking root exudates, microbes, and phosphorus (P) availability is established, this study provides new insights by offering a systematic quantification of these processes across distinct plant functional groups (grass, shrubs, and trees, as well as legumes and non-legumes) specifically within karst lithosols. We specifically clarify the divergent physiological strategies and the varying efficiencies of organic acid exudation that distinguish these functional groups. This high-resolution, comparative approach allows for a precise understanding of how low successional stages and plant types differentially recruit AMF and *phoD*-harboring bacteria to overcome P limitation, a detail previously lacking for this ecosystem. Particularly in karst regions of southwest China, selecting functional groups with high organic acid exudation capacity or regulating their rhizosphere microbial networks to improve P use efficiency has become a critical issue in ecological restoration practices. Therefore, investigating the root organic acid exudation characteristics of different plant functional groups and their associations with the synergistic P solubilization of AMF and *phoD*-harboring bacteria holds significant theoretical and practical importance for revealing plant adaptation strategies in karst ecosystems and guiding the selection and management practices of restoration species.

To address this question, three dominant grass, four shrub, and six tree species from grassland (G), shrubland (SH), and forest (T) restoration sites, respectively, in a karst ecosystem in southwestern China were classified into the grass–shrub–tree functional groups, and legume and non-legume functional groups. The C, N, and P contents of leaves and roots; the exudation rates of oxalic, citric and acetic acids; the contents of oxalic, citric and acetic acids, total P (TP), available P (AP), and microbial biomass P (MBP); and the activities of acid phosphatase (ACP) and alkaline phosphatase (ALP) in rhizosphere soil were measured, as well as the relative abundance, community structure, diversity, and co-occurrence network patterns of AMF and *phoD*-harboring bacteria. We hypothesize that: (1) oxalic acid dominates across different functional groups, though its exudation rate varies among them; (2) differences in organic acid composition are associated with the differentiation of rhizosphere AMF and *phoD* bacterial community structures; and (3) organic acids are linked to increased P availability via their associations with microbial networks and enzyme activity, with stronger synergistic effects observed in legumes. This study aims to achieve the following objectives: (1) to investigate the differences in organic acid exudation rates among different plant functional groups in the karst ecosystem, and (2) to elucidate the pathway by which different functional groups increase P availability through organic acid-mediated interactions between AMF and *phoD*-harboring bacteria.

## 2. Materials and Methods

### 2.1. Study Site

The study was conducted at the Huanjiang Karst Ecosystem Observation and Research Station of the Chinese Academy of Sciences (108°18′55.2″~108°19′56.7″ E; 24°43′57.6″~24°44′51.8″ N). The station encompasses an area of 146 hm^2^, with 7.5 hm^2^ of farmland predominantly distributed in the valley [39]. This region is characterized by a tropical monsoon climate, with a mean annual temperature ranging from 16.5 °C to 18.5 °C and precipitation ranging from 1400 to 1500 mm. The lowest temperatures are recorded in January (approximately 10.0 °C), and the highest in July (approximately 28.0 °C). The rainy season mainly occurs from April to September, while the dry season extends from October to March of the following year [40]. The study site features a typical peak-cluster depression landscape. According to the FAOUNESCO Soil Classification System, the soil type is categorized as Lithosol [41].

**Figure 1:**
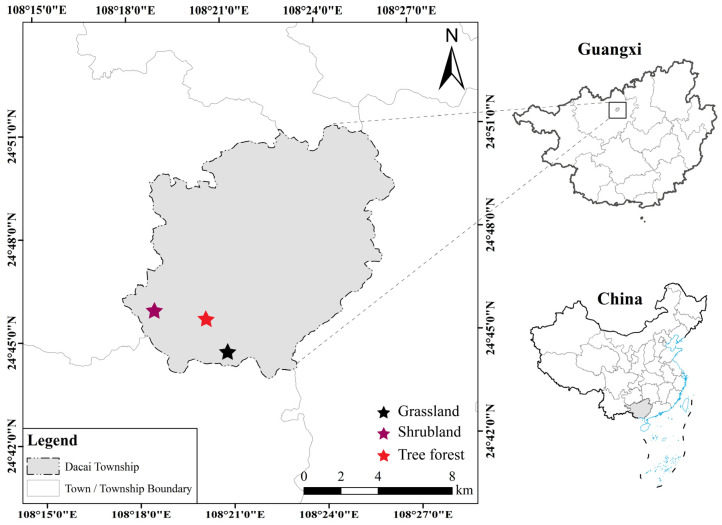
Geographical location and spatial distribution of the sampling plots in Hechi, southwestern China.

In June 2022, three stages of karst vegetation restoration (grassland (G), shrubland (SH), and forest (F)) were selected at this site (Figure 1). The G stage, occurring 15–18 years post-abandonment and typically located in depressions and on lower slopes, is characterized by a grass monoculture overwhelmingly dominated by *Miscanthus floridulus*. This stage exhibits low vegetation coverage, a thin litter layer, and limited surface biomass accumulation, with associated species such as *Imperata cylindrica*, *Caryopteris incana*, *Neyraudia reynaudiana*, and *Pueraria lobata*. Over time, the community transitions into the SH stage after 25–30 years, found in similar topographic positions of depressions and low-to-middle slopes. This stage is defined by a mixed shrub community co-dominated by *Vitex negundo* and *Pyracantha fortuneana*. Other prevalent shrubs include *Ligustrum sinense*, *Phanera championii*, *Pterolobium punctatum*, *Alchornea trewioides*, *Croton lachnocarpus*, *Zanthoxylum echinocarpum*, and *Alangium chinense*. The F stage, beyond 40 years of recovery, is dominated by *Zenia insignis* and *Cyclobalanopsis glauca*. It features a significantly thickened litter layer (>8 cm), well-developed understory vegetation, and a diverse species assemblage including *Mallotus philippensis*, *Cornus macrophylla*, *Cipadessa cinerascens*, *Machilus nanmu*, *Radermachera sinica*, *Cladrastis platycarpa, Gleditsia sinensis*, *Ligustrum lucidum*, *Cryptocarya chinensis*, *Myrsine kwangsiensis*, and *Vitex negundo* var. *cannabifolia*. All study plots for these stages were uniformly established on lower slope positions [39].

Three independent plots, each with an area of 20 m × 20 m, were established for each restoration stage. To avoid pseudoreplication, data from four selected dominant plant individuals per species within each 20 m × 20 m plot were collected and averaged. The resulting plot mean (*n* = 3 per vegetation stage) was used as the primary statistical unit for stage-wise comparisons to ensure statistical independence. Following the vegetation survey, the dominant species in each stage were identified, as presented in Table 1. Thus, there were three grass species in the grassland stage, four shrub species in the shrubland stage, and six tree species in the forest stage.

### 2.2. Sampling

#### 2.2.1. Collection of Root Exudated Organic Acids

**Figure 2:**
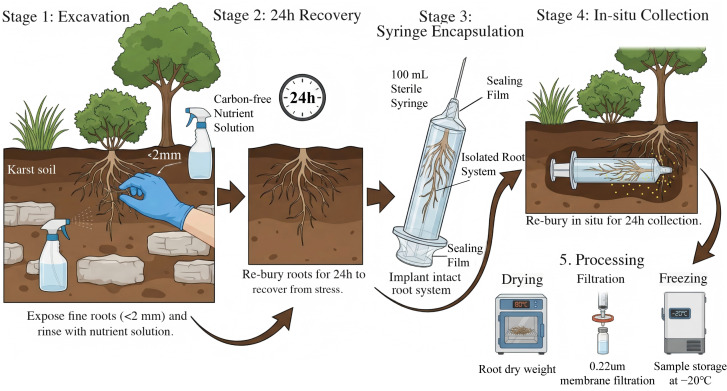
Graphic illustration of the conceptual workflow for the in situ root exudate collection procedure in the karst ecosystem. The diagram depicts the five discrete stages: (1) excavation of terminal roots while maintaining connectivity to the parent plant; (2) a 24 h soil recovery phase; (3) encapsulation of the intact, isolated root system within a sterile 100 mL syringe submerged in nutrient solution; (4) in-suit accumulation; and (5) laboratory processing.

Root exudates were collected in situ in June 2022 for all 13 dominant plant species [42], encompassing the grass (G), shrub (SH), and tree (T) functional groups, as well as the legume (L) and non-legume (NL) groups. For each species, four dominant individuals were selected for sampling. The standardized collection process for all functional groups was performed using a modified non-destructive syringe-encapsulation method (Figure 2). Roots with terminal diameters < 2 mm were gently excavated and immediately flushed with a carbon-free nutrient solution (KH_2_PO_4_, NH_4_NO_3_, MgSO_4_, K_2_SO_4_, and CaSO_4,_ Sinopharm Chemical Reagent Co., Ltd., Shanghai, China) to alleviate osmotic stress. The intact root system remained connected to the parent plant throughout the entire procedure to maintain physiological integrity. These roots were then re-buried in the original soil for a 24 h recovery period to mitigate potential excavation stress.

Following recovery, the cleaned, attached root system was carefully inserted into the barrel of a 100 mL sterile syringe. The syringe was filled with exactly 100 mL of carbon-free nutrient solution to submerge the roots, and both ends were hermetically sealed with sealing film to prevent leakage or external soil contamination. The syringes were re-buried in their original soil positions for 24 h of in situ accumulation. After this period, the solution was carefully retrieved, and its final volume was recorded. This final measurement was critical to account for any transpirational water uptake by the roots or localized seepage during the incubation period, ensuring the accuracy of subsequent concentration calculations. For the extensive and powerful multi-branching root systems of shrubs and trees, specific representative terminal fine root branches were selected to ensure the technique’s feasibility within the 100 mL syringe volume. Following collection, the roots were severed at the syringe entrance, dried at 80 °C to constant weight, and weighed to normalize the exudation rates. Replicates from three separate syringe locations per individual were combined into a 300 mL sterile bottle. The solution was sterilized by passing through a 0.22 μm filter membrane and temporarily stored in an incubator before being transported to the laboratory for storage at −20 °C [43]. The total sample size for root exudates was thirty-nine, reflecting thirteen species with three replicates each.

#### 2.2.2. Collection of Rhizosphere Soil, Fine Roots, and Leaves

Rhizosphere soil was defined as the soil fraction remaining adhered to the root surface after the loose bulk soil was removed by gentle shaking [43]. Following the collection of root exudates, fine roots and their surrounding soils (0–15 cm depth) were excavated. Soil tightly attached to the fine root surfaces was carefully collected using a brush [40]. Upon transport to the laboratory, all soil samples were passed through a 10-mesh sieve to remove visible impurities, such as stones and non-target plant debris. The homogenized soil was subsequently divided into specific portions for subsequent analysis: one portion was stored at −20 °C to be used for both soil organic acid determination and molecular-genetic studies, including total DNA extraction and microbial community sequencing of AMF and *phoD*-harboring bacteria. The remaining portions were either air-dried or processed through 20-mesh and 100-mesh screens for the determination of physicochemical properties and enzyme activities. Leaf and fine root samples were rinsed with ultrapure water, oven-dried at 65 °C to constant weight, and ground into a fine powder for elemental analysis.

### 2.3. Analysis

#### 2.3.1. Organic Acids in Soil and Root Exudates

The quantification of oxalic, citric, and acetic in root exudates and rhizosphere soil was performed using high-performance liquid chromatography (HPLC). All chemical reagents used in this study, including oxalic acid, citric acid, and acetic acid standards for HPLC analysis, were purchased from Sigma-Aldrich (St. Louis, MO, USA). For the analysis of root exudates, the collected liquid was initially filtered and subsequently concentrated into a powder via freeze-drying. The resulting residue was extracted with 5 mL of 0.1% phosphoric acid, shaken at 180 r/min for 30 min, and centrifuged at 10,000 r/min in a high-speed centrifuge for 10 min. The supernatant was passed through a 0.22 μm filter membrane prior to HPLC analysis (Agilent Technologies, Santa Clara, CA, USA) [44].

For rhizosphere soil, a 5 g freeze-dried sample was weighed into a 50 mL centrifuge tube and mixed with 10 mL of 0.1% phosphoric acid. The mixture was subjected to 30 min of shaking at 180 rpm followed by centrifugation at 10,000 rpm for 10 min. The supernatant was filtered through a 0.22 μm membrane, and the contents of oxalic, citric and acetic acids were determined using the Agilent 1260 HPLC system.

The chromatographic separation was achieved using an Agilent Hi-Plex H ion-exchange column (300 mm × 7.7 mm) with a mobile phase of 0.005 M H_2_SO_4_ at a constant flow rate of 0.6 mL/min. The column temperature was maintained at 55 °C, and organic acids were detected using a Diode Array Detector (DAD) (Agilent Technologies, Santa Clara, CA, USA) at a wavelength of 210 nm. Representative chromatograms and detailed calibration data are provided in the Supplementary Material.

All analytical calculations were based on external standards and purity specifications purchased from Sigma-Aldrich (St. Louis, MO, USA). The root exudation rates (*R*, μg·g^−1^·h^−1^) and soil organic acid contents (*C*, μg/g) were calculated using the following equations:

R=(c×V)/(w×t)

C=(c×V)/m

where *c* is the concentration of organic acids determined by HPLC (μg/mL), *V* is the total volume of the extract (mL), *w* is the root dry weight (g), *t* is the in situ collection duration (h), and *m* is the mass of the freeze-dried soil sample (g).

#### 2.3.2. General Soil and Plant Properties

(1) Soil Enzyme Activities

The activities of acid phosphatase (ACP) and alkaline phosphatase (ALP) were assayed using a fluorometric method. Sodium acetate buffer (50 mM, pH 5) and sodium bicarbonate buffer (50 mM, pH 8) were prepared for ACP and ALP measurements, respectively [41]. The substrate 4-methylumbelliferyl-phosphate (200 μm, Sigma-Aldrich) was used for both enzymes. In a 96-well microplate, 200 μL of soil suspension was combined with 50 μL each of standard solution, corresponding buffer, and substrate solution. The plates were incubated in the dark at 20 °C for 4 h, after which the reaction was terminated by adding 10 μL of 1.0 M NaOH per well. Fluorescence was immediately measured with a microplate fluorometer (Infinite M200 PRO, TECAN, Männedorf, Switzerland) using excitation/emission wavelengths of 365/450 nm.

(2) Nutrient Contents

Soil pH was determined potentiometrically using a glass electrode in a 1:2.5 soil-to-water (*w*/*v*) suspension. Available phosphorus (AP) was extracted using the Olsen method (0.5 M NaHCO_3_), which is specifically recommended for calcareous and alkaline soils common in karst regions to minimize the interference of high calcium content on P solubility [45]. A 2.5 g soil sample was shaken with 50 mL of extractant for 30 min, followed by quantification via the molybdenum-blue colorimetric method at 880 nm using UV-Vis spectrophotometry. Total phosphorus (TP) was measured using the same colorimetric method following soil digestion via sodium hydroxide fusion at 720 °C. Total nitrogen (TN) was determined using the semi-micro Kjeldahl digestion method and quantified with a flow injection analyzer (FIAstar 5000, FOSS, Hillerød, Denmark). For mineral nitrogen, ammonium nitrogen (NH_4_^+^-N) and nitrate nitrogen (NO_3_^−^-N) were extracted from fresh soil samples with 2 M KCl at a 1:5 soil-to-solution ratio, shaken for 1 h, and analyzed using the flow injection analyzer. Exchangeable calcium (Ca^2+^) and magnesium (Mg^2+^) were extracted with 1 M NH_4_OAc and quantified via an Atomic Absorption Spectrometry (AAS, PinAAcle 900T, PerkinElmer, Norwalk, CT, USA). The analysis followed the standard protocols described by Lu [46], with concentrations determined through multi-point calibration curves using certified reference standards.

Microbial biomass carbon (MBC), nitrogen (MBN), and phosphorus (MBP) were determined using the chloroform fumigation-extraction method. Refrigerated soil samples were homogenized and, if too dry, moistened with distilled water to 40% of water-holding capacity. They were then pre-incubated in sealed containers for 7–12 days, with 1 M NaOH traps to absorb CO_2_. After pre-incubation, soils were fumigated with chloroform for 24 h at 25 °C. MBC was extracted with potassium sulfate and measured using a TOC analyzer. MBN was extracted with potassium sulfate and determined by flow injection analysis (FIAstar 5000, FOSS, Eden Prairie, MN, USA). Soil MBP was extracted with sodium bicarbonate and measured by molybdenum-blue spectrophotometry [47].

For plant tissues, leaves and fine roots were rinsed with pure water, oven-dried at 75 °C to constant weight, ground, and passed through a 100-mesh sieve. The C and N contents of leaves and roots were analyzed using an elemental analyzer, while P content was determined by UV-Vis spectrophotometry after digestion.

#### 2.3.3. Illumina Sequencing Data Analysis

Total genomic DNA was extracted from 0.5 g of frozen soil using the FastDNA SPIN Kit for Soil (MP Biomedicals, Cleveland, OH, USA). The quality and quantity of the DNA were verified via agarose gel electrophoresis and UV spectrophotometry (NanoDrop Technologies, Wilmington, NC, USA) [47].

To characterize the total prokaryotic community, the V4 region of the 16SrRNA gene was amplified using the universal primers 515F (5′-GTGCCAGCMGCCGCGGTAA-3′) and 806R (5′-GGACTACHVGGGTWTCTAAT-3′). For functional bacterial communities, the *phoD* gene was amplified using primers ALPS-F730 (5′-CAGTGGGACGACCACGAGGT-3′) and ALPS-1101 (5′-GAGGCCGATCGGCATGTCG-3′) [44]. Raw sequences were processed, and *phoD* sequences were specifically annotated to the genus level using the RefSeq database to identify specific functional taxa across functional groups. Triplicate 25 μL PCR reactions were performed using the Ex Taq DNA Polymerase (Takara Bio USA, Inc., San Jose, CA, USA) [47].

The arbuscular mycorrhizal fungal (AMF) community was analyzed by targeting the Small Subunit (SSU) rRNA gene. The first round used primers NS31 (TTGGAGGGCAAGTCTGGTGCC) and AM1 (CTTTCCCGTAAGGCGCCGAA) [38]. The 20 µL reaction mixture contained 1 µL template DNA, 0.5 µL of each primer, 8 µL H_2_O, and 10 µL 2 × SYBR Premix Ex Taq (Takara Bio, Kusatsu, Shiga, Japan). The thermal profile was: 94 °C for 5 min; 35 cycles of 94 °C for 30 s, 45 °C for 58 s, 72 °C for 60 s; and a final extension at 72 °C for 10 min. The product from the first round was diluted 50-fold and used as the template for the second round. The second round used primers AMV4.60NF (AAGCTCGTAGTTGAATTTCG) and AMDGR (CCCAACTATCCCTATTAATCAT), with the same reaction mixture as the first round [38]. The thermal profile for the second round was: 94 °C for 3 min; 30 cycles of 94 °C for 45 s, 45 °C for 60 s, 72 °C for 60 s; and a final extension at 72 °C for 10 min. PCR products from both rounds were purified using the TIANquick Mini Purification Kit (Tiangen Biotech, Beijing, China).

Sequencing was performed by Magigene Biotechnology Co., Ltd. (Guangzhou, China). The sequencing data have been deposited in the NCBI database under BioProject accession numbers PRJNA1210578 (bacterial sequences) and PRJNA121052 (fungal sequences).

### 2.4. Statistical Analysis

Two classification schemes were employed to test distinct ecological hypotheses. Firstly, plants were categorized into grass, shrubs, and trees to examine how P acquisition strategies vary among functional groups [27]. Second, they were divided into legumes and non-legumes based on the hypothesis that high carbon cost of nitrogen fixation in legumes is associated with a more specialized organic acid strategy to alleviate P limitation [30].

Pearson correlations in SPSS 27 (2020) were used to analyze relationships among soil properties, microbial variables, P components, and organic acids across different functional groups. Differences among plant functional groups were evaluated using one-way ANOVA, followed by Tukey’s HSD post hoc test to determine significant differences at *p* < 0.05. All statistical units refer to plot-level means (*n* = 3) to avoid pseudoreplication. Heatmaps were generated using the “ggplot” and “RColorBrewer” packages in RStudio 4.5.1 (2024) to assess genus composition variability across samples. Alpha-diversity indices, including Shannon–Wiener, Simpson, ACE, and Chao1, were calculated using R 4.5.1 (2024). Specifically, the ‘vegan’ and ‘phyloseq’ packages within the RStudio 4.5.1 (2024) environment were employed to process the OTU/ASV tables generated from the Illumina sequencing data [48]. These tools are standard for assessing microbial richness and evenness in soil ecology studies. When constructing AMF and *phoD* co-occurrence networks, OTUs with relative abundances < 0.1% across all samples were excluded from analysis [44]. Co-occurrence networks were constructed using correlation coefficients of r > 0.6 and *p* < 0.05. Gephi was used to visualize co-occurrence networks for different functional groups [38]. The “graphics” package in R 4.5.1 (2024) was used to obtain node, edge, community count, average path length, degree, clustering coefficient, graph density, and modularity index for AMF network topology parameters [20]. Subsequently, the “Hmisc” package in R 4.5.1 (2024) was employed to generate an integrated correlation network diagram linking soil P, soil physicochemical properties, and bacteria harboring *phoD*/AMF genes at the genus level.

Mantel tests were conducted to examine relationships between diversity indices (Chao 1, Shannon–Wiener, Simpson, and evenness) and the *phoD*/AMF-carrying community structure, as well as soil nutrients and ALP/ACP activities. A random forest model (using the “randomForest” package in R 4.5.1 (2024)) was employed to identify the key predictors of soil AP and the community structure harboring *phoD*/AMF genes. To ensure model stability and optimize performance, the forest was grown with 500 trees (ntree = 500) and five variables randomly sampled at each split. The significance of each predictor was determined through 1000 permutations using the “rfPermute 4.5.1” package to validate that the identified importance rankings were robust and to minimize the risk of overfitting. The relative importance of measured variables was evaluated based on mean squared error (MSE%). Finally, statistical analysis using Structural Equation Modeling (SEM) was employed to examine directional associations between ALP, pH, AP, organic carbon, TN, and the relative abundance of *phoD*/AMF-carrying genes. The best-fitting model was selected based on model fit using maximum likelihood estimation, incorporating chi-square (χ^2^) tests, goodness-of-fit indices, and approximate root mean square error.

To rigorously disentangle the relative contributions of abiotic and biological drivers, both soil physicochemical properties and biological variables were included as independent predictors in the random forest and Structural Equation Modeling (SEM) analyses. This approach allowed for the identification of the most significant predictors of P availability while accounting for background abiotic variations. It is important to note that while Structural Equation Modeling (SEM) was used to test potential pathways, this study is observational in nature. Therefore, the SEM results identify statistical associations and directional links between variables rather than establishing direct cause-and-effect relationships.

## 3. Results

### 3.1. Soil P Fractions, Organic Acids, and Phosphatase Activities Across Functional Groups

**Figure 3:**
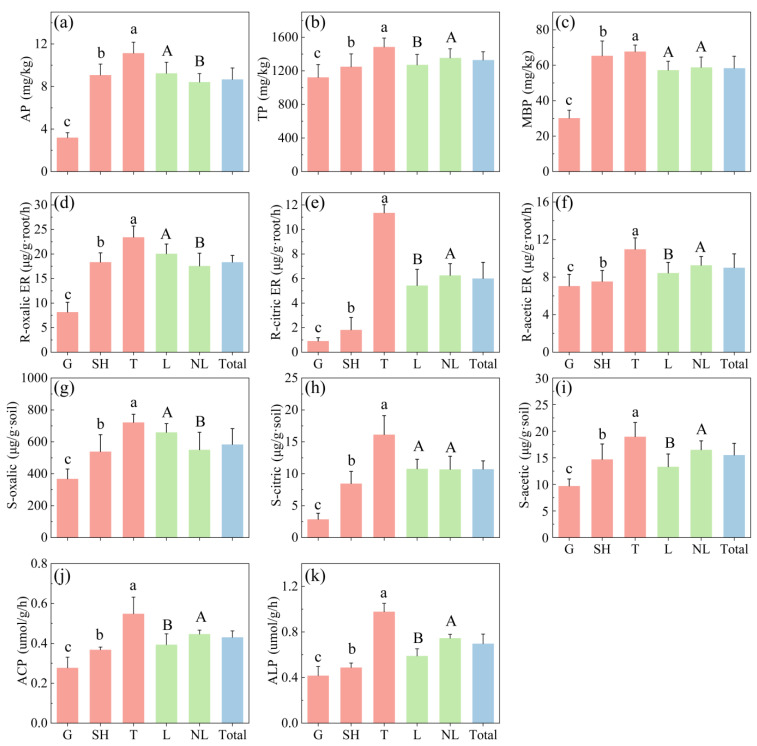
Content of P, organic acids, and phosphatase activities in the rhizosphere soil of functional group plants of grass shrub and trees. (**a**) AP, available P; (**b**) TP, total P; (**c**) MBP, microbial biomass P; (**d**) R-oxalic ER, root oxalic acid exudation rate; (**e**) R-citric ER, root citric acid exudation rate; (**f**) R-acetic ER, root acetic acid exudation rate; (**g**) S-oxalic, soil oxalic acid content; (**h**) S-citric, soil citric acid content; (**i**) S-acetic, soil acetic acid content; (**j**) ACP, acid phosphatase; (**k**) ALP, alkaline phosphatase. G, grass; SH, shrub; T, trees; L, leguminous plants; NL, non-leguminous plants. Vertical bars represent the standard error (SE) of the mean. Individual data points represent plot-level baseline (*n* = 3 for each group). The “Total” column signifies the ecosystem-level baseline, calculated as the overall mean of all 13 sampled plant species (*n* = 39). Different letters mean significant differences (*p* < 0.05) based on Tukey’s HSD test.

To evaluate the P acquisition strategies of various vegetation types, we compared specific functional groups against the ecosystem-level average. The rhizosphere soil of the tree group exhibited the highest levels of AP, TP, and MBP, as well as the highest ACP and ALP activities, followed by the shrub group. The grass group showed the lowest values for these parameters (Figure 3). The AP content of the legume group was significantly higher than that of the non-legume group. However, no significant differences in TP, MBP, ACP, or ALP were observed between these two groups.

Among the grass–shrub–tree functional groups, trees exhibited the highest rhizosphere soil organic acid content, followed by shrubs, with grass having the lowest levels. Among rhizosphere organic acids, oxalic acid was the most abundant. Tree root organic acid exudation rates were also significantly higher than those of shrubs and grass; the exudation rate of oxalic acid by plant roots was substantially higher than that of citric acid and acetic acid. In the legume and non-legume functional groups, oxalic acid content in the rhizosphere soil of legumes was significantly higher than that of non-legumes, but the trends for citric acid and acetic acid were opposite. Among the organic acids in the rhizosphere soil, oxalic acid content was the highest. The oxalic acid exudation rate from legume roots was also significantly higher than that from non-legume roots. Furthermore, among the organic acid exudation rates from plant roots, the oxalic acid exudation rate was the highest.

### 3.2. Community Structure, Diversity, and Co-Occurrence Networks of AMF and phoD-Harboring Bacteria Across Functional Groups

**Figure 4:**
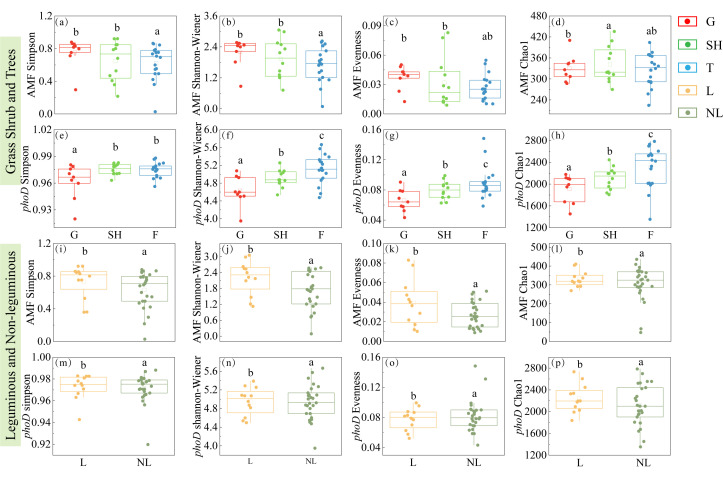
Diversity indices of AMF and *phoD* in different functional groups. (**a**) AMF Simpson, the Simpson index of AMF in grass shrub and trees; (**b**) AMF Shannon, the Shannon–Wiener index of AMF in grass shrub and trees; (**c**) AMF Evenness, the Evenness index of AMF in grass shrub and trees; (**d**) AMF Chao1, the Chao1 index of AMF in grass shrub and trees; (**e**) *phoD* Simpson, the Simpson index of *phoD *in grass shrub and trees; (**f**) *phoD* Shannon, the Shannon–Wiener index of *phoD *in grass shrub and trees; (**g**) *phoD* Evenness: the Evenness index of *phoD *in grass shrub and trees; (**h**) *phoD* Chao1, the Chao1 index of *phoD *in grass shrub and trees. (**i**) AMF Simpson, the Simpson index of AMF in leguminous and non-leguminous; (**j**) AMF Shannon, the Shannon–Wiener index of AMF in leguminous and non-leguminous; (**k**) AMF Evenness, the Evenness index of AMF in leguminous and non-leguminous; (**l**) AMF Chao1, the Chao1 index of AMF in leguminous and non-leguminous; (**m**) *phoD* Simpson, the Simpson index of *phoD *in leguminous and non-leguminous; (**n**) *phoD* Shannon, the Shannon–Wiener index of *phoD *in leguminous and non-leguminous; (**o**) *phoD* Evenness: the Evenness index of *phoD *in leguminous and non-leguminous; (**p**) *phoD* Chao1, the Chao1 index of *phoD *in leguminous and non-leguminous. G, grass; SH, shrub; T, trees; L, leguminous plants; NL, non-leguminous plants. Individual dots represent biological replicates from three independent plots (*n* = 3). In the boxplots, the horizontal center line represents the median value, while the box limits indicate the 25th and 75th percentiles. Vertical lines (whiskers) show the range of the data excluding outliers. Significant differences (*p* < 0.05) between groups are denoted by different lowercase letters.

**Figure 5:**
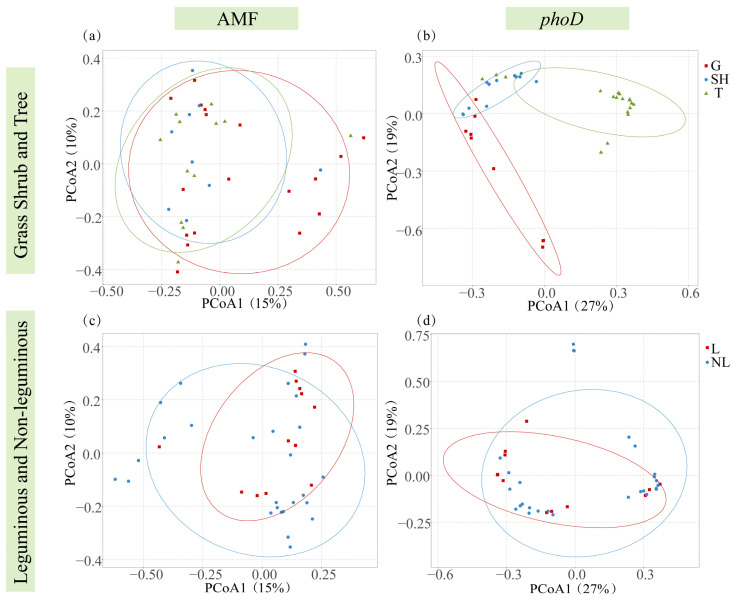
PCoA showing differences in *phoD* bacteria and AMF communities across different functional groups. (**a**) PCoA plot of AMF genes in the grass, shrub, and tree; (**b**) PCoA plot of *phoD* genes in the grass, shrub, and tree; (**c**) PCoA plot of AMF genes in the leguminous and non-leguminous; (**d**) PCoA plot of *phoD* genes in the leguminous and non-leguminous.

The Shannon–Wiener, Simpson, Evenness, and Chao 1 diversity indices for *phoD*-harboring bacteria progressively increased from grass to shrub to tree communities (Figure 4e–h), whereas AMF diversity indices peaked in shrub communities (Figure 4a–d). Additionally, no significant differences were observed in the Shannon–Wiener, Simpson, Evenness, and Chao 1 indices for *phoD*-harboring bacteria between legume and non-legume functional groups (Figure 4n–q). Correspondingly, AMF indices were higher in legumes than in non-legumes (Figure 4i–m). Simultaneously, PCoA results revealed significant compositional differences in AMF and *phoD*-harboring bacterial communities across plant functional groups (Figure 5).

**Figure 6:**
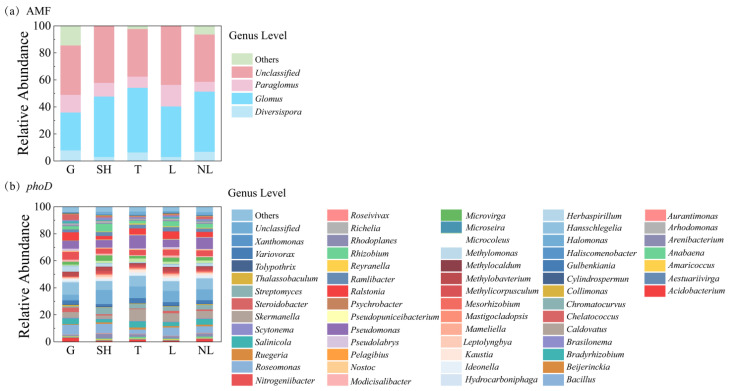
Changes in relative abundance of arbuscular mycorrhizal AMF and *phoD* at the genus level in different functional groups. (**a**) Relative abundance plot of AMF genes across two functional groups; (**b**) Relative abundance plot of *phoD* genes across two functional groups. G, grass; SH, shrub; T, trees; L, leguminous plants; NL, non-leguminous plants.

At the genus level (>5% relative abundance) for *phoD*-harboring bacteria, *Hansschlegelia* (9.07%) and *Bacillus* (7.25%) were the most prevalent in grass; *Halomonas* (10.29%), *Bacillus* (6.96%), and *Hansschlegelia* (6.19%) were major genera in the shrub group; and *Pseudomonas* (9.73%) and *Halomonas* (8.58%) were dominant in trees. Additionally, *Halomonas* (8.3%), *Hansschlegelia* (7.06%), and *Bacillus* (6.14%) were dominant in leguminous plants, whereas *Hansschlegelia* (7.99%), *Halomonas* (7.92%), and *Bacillus* (4.87%) were dominant in non-leguminous plants (Figure 6b). At the order level (>4% relative abundance), Hyphomicrobiales (30.14%, 28.39%, and 25.11% for grass, the shrub group, and trees, respectively) and Burkholderiales (30.14%, 28.39%, and 25.11%, respectively) were dominant in grass, shrub group, and trees. Hyphomicrobiales (28.34% and 26.81%), Burkholderiales (12.97% and 10.36%), and Oceanospirillales (10.78% and 9.1%) were dominant in leguminous and non-leguminous plants, respectively (Figure S1b).

For AMF at the genus level (>5% relative abundance), *Glomus* (28.04%, 44.77%, and 47.81%), *Paraglomus* (13.09%, 10.11%, and 8.29%), and *Diversispora* (7.93%, 3.01%, and 6.43%) were dominant in grass, the shrub group, and trees, respectively. *Glomus* (37.43% and 44.48%), *Paraglomus* (15.94% and 7.3%), and *Diversispora* (3.02% and 6.93%) were dominant in leguminous and non-leguminous plants, respectively (Figure 6a). At the order level (>4%), Glomerales (28.37%, 47.87%, and 47.89%), Paraglomerales (22.87%, 18.31%, and 10.85%), and Diversisporales (24.39%, 5.55%, and 9.95%) were dominant in grass, the shrub group, and trees, respectively; while Glomerales (37.34% and 46.06%), Paraglomerales (23.04% and 12.76%), and Diversisporales (18.41% and 9.04%) were dominant in leguminous and non-leguminous plants, respectively (Figure S1a).

**Figure 7:**
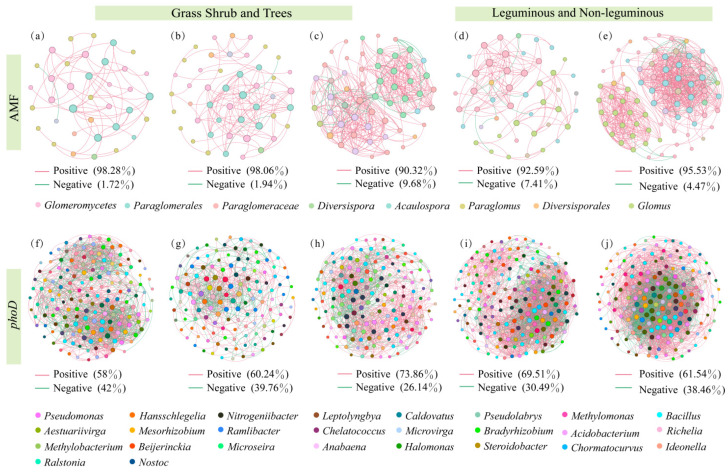
Co-occurring network analysis of AMF and *phoD* communities in different functional groups at the genus level. The red line represents positive correlation, while the green line represents negative correlation. (**a**) AMF taxonomic units of grass; (**b**) AMF taxonomic units of shrub; (**c**) AMF taxonomic units of trees; (**d**) AMF taxonomic units of leguminous plants; (**e**) AMF taxonomic units of non-leguminous plants; (**f**) *phoD* classification units of grass; (**g**) *phoD* classification units of shrub; (**h**) *phoD* classification units of trees; (**i**) *phoD* taxonomic units of leguminous plants; (**j**) *phoD* taxonomic units of non-leguminous plants.

At the genus level, co-occurrence networks revealed that tree AMF communities exhibited the highest complexity and compactness, followed by shrubs, while grass AMF communities showed the lowest complexity and compactness. Non-legume AMF communities demonstrated higher compactness and complexity than legume AMF communities (Figure 7). Within the grass–shrub–tree functional groups, the AMF gene–microbe networks comprised 36, 47, and 57 nodes with 58, 103, and 372 edges, respectively. In the legume–non-legume functional groups, these networks contained 48 and 76 nodes with 108 and 671 edges. Furthermore, both the average degree and average clustering coefficient were highest in the tree stage, followed by the shrub stage, and lowest in the grass stage. Within the legume and non-legume functional groups, non-legumes exhibited higher values than legumes. Furthermore, the positive correlation rates of AMF co-occurrence networks across functional groups were 98.28% (grass), 98.06% (shrub), 90.32% (tree), 92.95% (legume), and 95.53% (non-legume).

**Figure 8:**
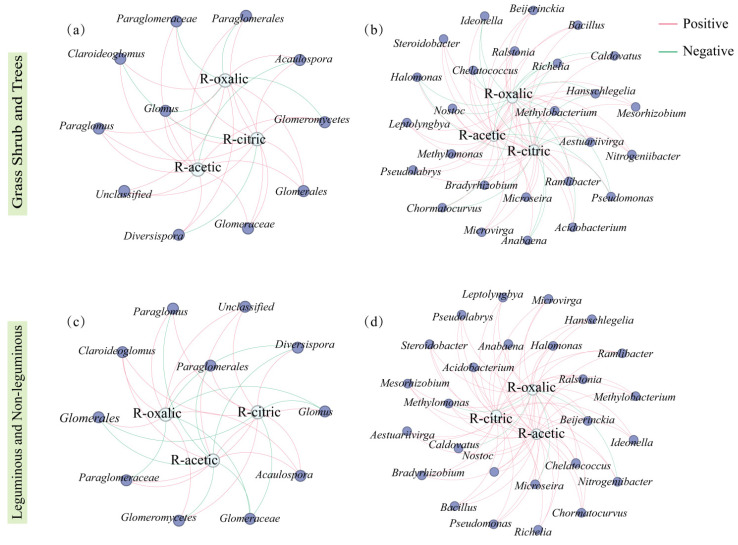
At the genus level, the correlation network of *phoD*-containing bacteria and AMF with soil organic acids. (**a**) correlation network of AMF with soil organic acids in the grass, shrub, and tree; (**b**) correlation network of *phoD*-containing bacteria with soil organic acids in the grass, shrub, and tree; (**c**) correlation network of AMF with soil organic acids in the leguminous and non-leguminous; (**d**) correlation network of *phoD*-containing bacteria with soil organic acids in the leguminous and non-leguminous. The green edge indicate negative interactions between two nodes, and the red edge indicate positive interactions between two nodes.

The *phoD* community of trees exhibited the highest complexity and compactness, followed by grass, while shrubs showed the lowest complexity and compactness. Non-legume *phoD* communities demonstrated higher compactness and complexity than legume *phoD* communities (Figure 8). Within the grass–shrub–tree functional groups, the *phoD* gene microbial networks comprised 160, 133, and 159 nodes, respectively, with 1431, 576, and 1232 edges. In the legume and non-legume functional groups, the networks contained 178 and 167 nodes, respectively, with 2276 and 3450 edges. Secondly, both the average degree and average clustering coefficient were highest in the tree stage, followed by the grass stage, and lowest in the shrub stage. Within the legume–non-legume functional groups, non-legumes exhibited higher values than legumes. Additionally, the positive correlation in *phoD* co-occurrence networks across both functional groups were 58% (grass), 60.24% (shrub), 73.86% (tree), 69.51% (legume), and 61.54% (non-legume).

Network analysis of organic acids with AMF and *phoD* revealed that in the grass–shrub–tree functional groups, oxalic acid exudation and citric acid exudation positively correlated with *phoD*-containing bacteria such as *Halomonas* and *Bacillus*, as well as with mycorrhizal fungi *Glomus* and *Paraglomus* (Figure 8a,b). In legume and non-legume functional groups, organic acids from root exudation showed positive correlations with mycorrhizal fungi *Glomus*, *Paraglomus*, and *Diversispora*, as well as with *phoD*-containing bacteria such as *Halomonas* and *Hansschlegelia*.

### 3.3. Associations of Organic Acids and Microbes with P Availability

**Figure 9:**
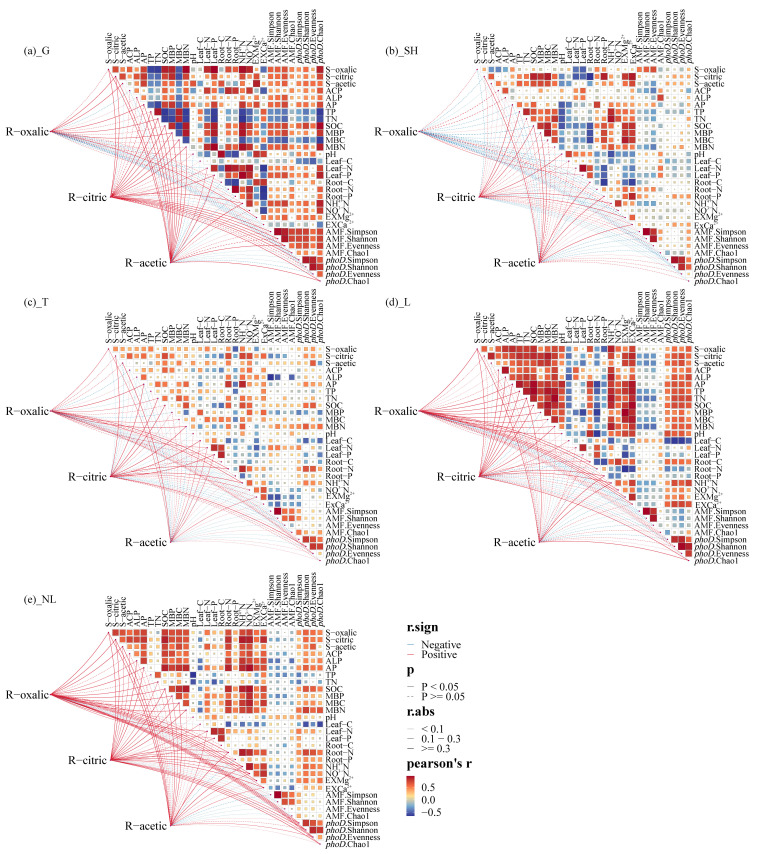
Correlation analysis between organic acids and soil properties. R-oxalic, oxalic acid of root exudates; R-citric, citric acid of root exudates; R-acetic, acetic acid of root exudates; S-oxalic, soil oxalic acid; S-citric, soil citric acid; S-acetic, soil acetic acid; ACP, acid phosphatase; ALP, alkaline phosphatase; AP, available phosphorus; TP, total phosphorus; TN, total nitrogen; SOC, soil organic carbon; MBP, microbial biomass phosphorus; MBC, microbial biomass carbon; MBN, microbial biomass nitrogen; pH, potential of hydrogen; Leaf-C, C contents of leaf; Leaf-N, N contents of leaf; Leaf-P, P contents of leaf; Root-C, C contents of root; Root-N, N contents of root; Root-P, P contents of root; NH^4+^N, ammonium N; NO^3−^N, nitrate N; AMF Simpson, the Simpson diversity index of AMF; AMF Shannon, the Shannon–Wiener diversity index of AMF; AMF Evenness, the Evenness index of AMF; AMF Chao1, the Chao1 index of AMF; *phoD* Simpson, the Simpson index of *phoD*; *phoD* Shannon, the Shannon–Wiener index of *phoD*; *phoD* Chao1, the Chao1 index of *phoD*; *phoD* Evenness: the Evenness index of *phoD*. G, grass; SH, shrub; T, trees; L, leguminous plants; NL, non-leguminous plants.

Mantel test results indicate that in grass, oxalic acid exudation and citric acid exudation exhibit significant positive correlations with TP, MBP, and AP; in shrub communities, citric acid exudation showed significant positive correlations with TP, MBP, and AP, while oxalic acid exudation and citric acid exudation exhibited significant negative correlations with AP and TP. Finally, in forest communities, R-oxalic acid showed significant positive correlations with TP, MBP, and AP. Leguminous plants exhibited significant positive correlations between oxalic acid exudation and TP, MBP, and AP, while citric acid exudation showed a significant negative correlation with TP. In contrast, non-leguminous plants demonstrated significant positive correlations between oxalic acid exudation and citric acid exudation with TP, MBP, and AP, and citric acid exudation showed a negative correlation with TP (Figure 9). Therefore, oxalic is a key factor associated with soil P components.

**Figure 10:**
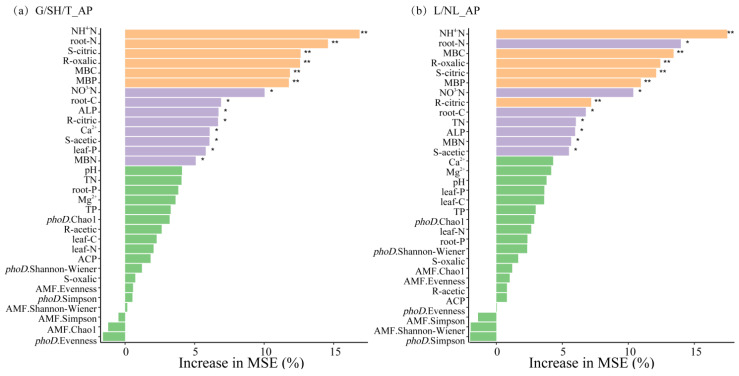
Ranking of important factors affecting P availability in different functional groups. G, grass; SH, shrub; T, trees; L, leguminous plants; NL, non-leguminous plants. ** *p* < 0.01; * *p* < 0.05.

**Figure 11:**
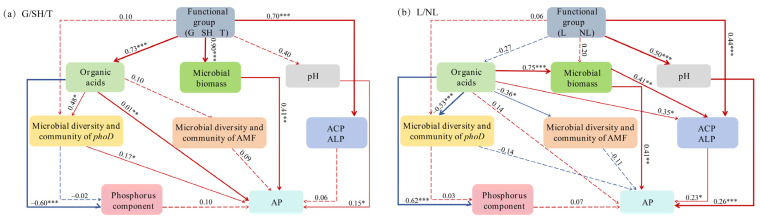
Structural Equation Modeling of *phoD* diversity, soil nutrients, phosphatase activity, P components, organic acids, and AMF on available P among different functional groups. G, grass; SH, shrub; T, trees; L, leguminous plants; NL, non-leguminous plants. The parameters of these models: (**a**) X_2_ = 1.741, degrees of freedom = 3, *n* = 39, GFI = 0.947, AGFI = 0.816, *p* = 0.661, RMSEA = 0.000; (**b**) X_2_ = 1.376, degrees of freedom = 3, *n* = 39, GFI = 0.981, AGFI = 0.896, *p* = 0.909, RMSEA = 0.000. Blue represents negative impacts, and red represents positive impacts. Solid lines indicate statistically significant paths, and dashed lines indicate non-significant relationships. The numbers adjacent to the arrows are standardized path coefficients, representing the relative strength of each effect. *** *p*< 0.001, ** *p* < 0.01, * *p* < 0.05.

The random forest model results indicate that several factors are significant predictors of AP within the grass–shrub–tree functional groups, including NH4-N, root-N, soil citric acid, oxalic acid exudation, microbial biomass (MBC, MBP), NO3-N, root-C, ALP, citric acid exudation, Ca^2+^, and soil citric acid (Figure 10a). For leguminous and non-leguminous functional groups, important predictors included NH4-N, root-N, MBC, oxalic acid exudation, soil citric acid, MBP, NO^3−^N, citric acid exudation, root-C, TN, ALP, MBN, and soil acetic acid (Figure 10b). Based on the SEM analysis results (Figure 11), within the grass–shrub–tree functional groups, organic acids and microbial biomass were significantly associated with AP. Vegetation was linked to higher AP through its associations with organic acids, microbial biomass, and phosphatase activity. Additionally, organic acids are linked to higher AP via higher soil phosphatase content associated with shifts in *phoD* and AMF diversity and abundance. In the Structural Equation Model for the legume–non-legume functional group, organic acids, microbial biomass, pH, and phosphatase activity were positively associated with AP. However, organic acids ultimately reduced AP content by decreasing *phoD* and AMF diversity and community abundance. Furthermore, the functional group was significantly linked to AP via its association with phosphatase activity and microbial biomass, while the minor variations in pH played a negligible role in phosphorus mobilization compared to organic acid chelation.

## 4. Discussion

### 4.1. Differences in Organic Acid Exudation Rates Among Plant Functional Groups

In this study, both the exudation rate and soil content of organic acids increased from the grass group to the shrub group and then to the tree group (Figure 1). Several factors may explain this phenomenon. (1) Higher biomass entails a greater demand for nutrients. Research indicates that during the vegetation restoration process in karst ecosystems, plant biomass exhibits a significant upward trend [8]. Plants with higher biomass have a greater P demand, as evidenced by the increasing P limitation in later-stage tree forests, indicated by the increase in leaf N:P ratio from 14.2 in grass to 28.6 in trees. To acquire more P, plants exhibit higher exudation of organic acids, which is linked to an increase in available P [44]. In our study, trees exhibited significantly higher organic acid exudation rates and higher contents of oxalic and citric acids in the rhizosphere soil compared to the shrub group and the grass group, and these parameters were significantly positively correlated with soil available P. These findings support this notion. (2) A greater abundance of symbiotic and free-living microbes requires more carbon sources [49]. Accordingly, microbial biomass P—a proxy for microbial population size—increased significantly from the grass and shrub groups to the tree group (Figure 3c), consistent with the elevated carbon demand (Figure 3c), corresponding to an increased carbon demand. Microbes utilize carbon sources in exchange for available P: the AMF hyphal network consumes 20–30% of the host’s photosynthetic carbon, while *phoD* bacteria require 15.8 μg of carbon to synthesize one unit of phosphatase [50]. In return, AMF expand the P absorption range via its hyphal network, and *phoD* bacteria mineralize organic P by the exudation of ALP. Their synergistic interaction enhances soil P availability, ultimately providing P nutritional feedback to the plant.

Furthermore, legumes showed significantly higher root organic acid exudation rates and soil oxalic acid content than non-legumes, whereas the opposite trend was observed for citric acid and acetic acid. Potential reasons for this observation include the following: First, the high carbon cost of symbiotic nitrogen fixation is associated with the prioritization of oxalic acid exudation in legumes. The biological nitrogen fixation process in legume root nodules consumes 6~12 g of carbon for every gram of nitrogen fixed [31], forming a massive carbon sink that competes with organic acid synthesis for photosynthetic products. Notably, the biosynthetic pathways of different organic acids exhibit significant variations in carbon requirements: oxalic acid synthesis demands only two carbon atoms, whereas citric acid and acetic acid require higher metabolic energy investment per molecule [51]. Under carbon-limited conditions imposed by nodule metabolism, legumes prioritize oxalic acid synthesis. In contrast, non-legumes, free from the carbon costs of symbiotic nitrogen fixation, exhibit fundamentally different strategies for allocating photosynthetic products. Specifically, non-legumes can direct more carbon resources toward synthesizing diverse organic acids rather than focusing primarily on oxalic acid [36]. Second, positive feedback loops between oxalic acid and specific microbial communities reinforce legume exudation patterns. Flavonoid signaling molecules released by legume roots attract specific microbial communities, including *Halomonas* species possessing high-affinity oxalic acid transporters and metabolic enzymes [38]. Recruited *Halomonas* utilize oxalic acid as a carbon source, proliferate extensively in the rhizosphere, and release highly active ALP via exudation. This ALP mineralizes organic P, releasing available P that alleviates plant P deficiency and supports sustained oxalic acid exudation, thereby forming an oxalic acid-associated positive feedback loop. Furthermore, *Halomonas* acidifies and dissolves residual calcium–phosphate complexes, further enhancing P availability. In contrast, mixed organic acids exuded by non-legumes lack potent flavonoid signals, resulting in recruited *phoD* bacterial communities that are more diverse but less specialized. Notably, oxalic acid exudation rates in legume rhizosphere show a significant positive correlation with *Halomonas* abundance (Figure 8), strongly supporting this microbe-mediated enhancement mechanism.

In this study, oxalic accounted for 63.1~79.8% of the total organic acid concentration in rhizosphere soil, and the exudation rate of oxalic acid by plant roots was significantly higher than that of citric acid and acetic acid. This finding indicates that oxalic acid is the primary organic acid exuded by all plant functional groups. This may be attributed to oxalic acid synthesis requiring only 50% of the carbon expenditure of citric acid, while achieving 2.2 times higher P release efficiency per unit carbon, thereby optimizing resource allocation under P limitation. This supports the second hypothesis. It is important to note that while our study highlights oxalic acid as a primary factor associated with P availability, alternative interpretations in soil ecology often favor tricarboxylic acids like citric acid due to their higher carboxyl group density and superior metal-chelating potential. In many non-karst calcareous systems, citric acid is reported to be significantly more effective at P solubilization than dicarboxylic acids. However, our data suggest that in the specific context of karst lithosols, the high carbon efficiency and calcium affinity of oxalic acid represent a more viable trade-off for plants facing extreme carbon and P limitations. This indicates that the ‘oxalic-dominant’ model may be an environment-specific adaptation rather than a universal rule for all calcareous soils.

### 4.2. Microbial Differences in the Rhizosphere Soil of Different Plant Functional Groups

This study reveals the significant associations between plant root exudates, particularly oxalic, and the community structure and interactions of AMF and *phoD*-harboring bacteria across the two different functional grouping schemes. This organic acid-linked microbial synergy is a primary factor associated with soil P availability.

Within the grass–shrub–tree functional group, *phoD*-harboring bacterial diversity increased from trees to shrubs to grass (Figure 4e–h). This phenomenon likely arises because the continuous oxalic acid exudation from tree roots provides a stable carbon source for oxalic-metabolizing *phoD*-harboring bacteria (*Pseudomonas* and *Halomonas*), thereby enriching these functional bacterial genera [4]. While the relative abundance of these *phoD*-harboring genera ranged from 6% to 10.3%, they represented the highest-ranking functional groups within the highly diverse bacterial community, where the majority of other taxa remained below 0.1%. However, AMF diversity peaked during the shrub stage (Figure 4a–d). This likely stems from a trade-off between rapid P acquisition and the establishment of stable symbiotic networks during the shrub phase, prompting plants to invest more carbon resources to recruit diverse AMF species [11]. During the tree stage, the system stabilizes and is dominated by a few highly efficient fungal species (*Glomus*). This may be due to direct carbon support from thickening litter layers, where enhanced mycelial expansion capacity significantly improves mycorrhizal P uptake efficiency [6]. Legumes exhibit the highest oxalic exudation rates and AMF diversity, likely reflecting a specialized carbon investment strategy to reconcile the trade-off between nitrogen fixation/phosphorus consumption and P uptake.

Within the legume and non-legume functional groups, legumes exhibited significantly higher AMF diversity than non-legumes, likely due to isoflavones from root exudation attracting high-affinity *Glomus* strains [51]. Although the abundance of *Glomus* in legumes was slightly lower than in non-legumes, its strong positive correlation with theoxalic acid exudation rate indicates that oxalic acid plays a key role in influencing *Glomus* populations. Non-legumes relied on multi-species collaboration to address P limitation, exhibiting higher AMF network complexity. Among *phoD*-harboring bacteria, although legume and non-legume diversity showed no significant difference, genus-level composition differed markedly: legumes showed a statistically significant preference for *Halomonas*, while *Hansschlegelia* was more closely associated with non-legumes. Previous studies indicate that legume-associated *Halomonas* strains exhibit ALP activity 2.3 times higher than common strains [33]. Legume microbial networks exhibited 69.5% positive-linked interactions, exceeding the 61.5% in non-legumes, further illustrating how specialized interactions are linked to higher functional efficiency as an ecological strategy.

Network analysis revealed that from herbaceous to arboreal stages, both communities exhibited significant increases in node count, connectivity, and complexity (Figure 7 and Figure 8), indicating increasingly tight microbial interactions. The tree-stage *phoD* network exhibited a remarkably high 73.9% positive correlation rate (Figure 8), indicating that root exudation of large amounts of oxalic acid fosters a cooperative microbial environment in the rhizosphere. Within this environment, AMF hyphae facilitate the spread and colonization of *phoD* bacteria, while *phoD* bacteria empower the AMF hyphal network through their ability to mineralize organic P. Thus, organic acids further strengthen the interaction network between AMF and *phoD* bacteria.

### 4.3. Associations Between Organic Acids and Increase P Availability by AMF and phoD-Harboring Bacteria

Structural Equation Modeling and random forest analysis jointly confirm that oxalic acid is associated with the alleviation of soil P limitation through direct chemical activation and indirect links to microbial community interactions (Figure 10 and Figure 11). The direct chemical pathway was validated by multiple statistical results: as organic acid exudation increased from the shrub to tree stages, exchangeable Ca^2+^ and available P in the rhizosphere rose synchronously (Figure 3), consistent with the Ca^2+^ chelation mechanism dissolving Ca-P complexes. Oxalic acid, leveraging its high calcium chelation capacity [17], forms stable calcium oxalic acid precipitates, thereby releasing P from the dominant insoluble calcium–P fraction in karst soils. Concurrently, soil pH remained relatively stable across restoration, varying by only 0.05 units. While abiotic factors such as soil pH and exchangeable Ca^2+^ are known to influence P solubility, our results suggest they are not the primary factors associated with the observed P availability shifts in this system. Our SEM analysis demonstrates that minor variations in pH played a negligible role in phosphorus mobilization compared to the robust effects of organic acid chelation and phosphatase activity. This suggests that in these alkaline karst soils, P activation is linked primarily to the calcium chelation pathway of oxalic acid, which releases P by forming stable calcium, rather than through direct acidification [52].

Beyond direct activation, organic acids shaped the rhizosphere microbial community as a carbon source, establishing an indirect pathway for P acquisition. Organic acids differentially enriched specific arbuscular mycorrhizal fungi and *phoD*-carrying bacteria across functional groups. During grass–shrub–tree succession, elevated oxalic acid concentrations correlated with increased relative abundances of the arbuscular mycorrhizal fungus *Glomus*, and the bacteria *Pseudomonas* and *Bacillus* (Figure 6), reflecting their capacity to utilize organic acids as carbon sources. In legumes, high oxalic acid environments selectively enriched *Halomonas*—a genus possessing high-affinity oxalic acid transporters and oxalic acid decarboxylase [53]. Network analysis revealed that organic acids reshaped interactions among these microbial communities: both AMF and *phoD* network complexity progressively increased from the herbaceous to the tree stage (Figure 7 and Figure 8), indicating that higher organic acid exudation promoted the formation of more complex microbial networks. The *phoD* network at the tree stage exhibited a significantly higher positive correlation rate (Figure 8), indicating that sustained oxalic acid input fosters a cooperative rather than competitive microbial environment: AMF hyphae promote *phoD* bacterial colonization, while *phoD* bacteria mineralize organic P into orthophosphate for efficient uptake by AMF hyphae [32,33].

The functional outcome of this microbial community is enhanced phosphatase activity. Random forest models confirmed that ALP activity is a significant predictor of AP (Figure 10), while analysis via the Structural Equation Model (SEM) analysis demonstrated that *phoD* diversity is positively associated with AP via links to phosphatase activity (Figure 11). Notably, legume-enriched *Halomonas* strains exhibited 2.3-fold higher ALP activity than common strains [54], converting organic acid carbon investment into efficient P mineralization. Higher positive interaction frequencies within legume microbial networks (Figure 8) indicate that specialized interactions enhance functional efficiency.

Direct and microbial pathways exhibit systematic differences across functional groups. In oxalic acid-dominant tree and legume communities, the direct chelation pathway is particularly crucial, as evidenced by the strong correlation between oxalic acid, calcium ions, and ALP activity (Figure 9). Concurrently, these communities exhibit highly developed microbial networks and robust phosphatase activity, indicating stable microbial pathway operation. In shrub and non-legume communities, high citric acid dependency correlates more closely with Fe-P/Al-P activation, accompanied by higher microbial community diversity but lower specificity.

Furthermore, the total pool of rhizosphere organic acids likely represents a synergistic contribution from both plant roots and recruited microorganisms [13,16]. Many dominant *phoD*-harboring bacteria identified in this study, such as *Pseudomonas* and *Bacillus*, are well-documented plant growth-promoting rhizobacteria (PGPR) that can actively secrete biogenic organic acids in addition to phosphatases [19,55]. Similarly, rhizosphere micromycetes, including certain arbuscular mycorrhizal fungi (AMF) like *Glomus*, may further supplement the rhizosphere acid pool to enhance P mobilization [56,57]. This suggests a positive feedback loop: root-exuded oxalic acid provides the initial carbon source to support microbial proliferation, which in turn is linked to secondary microbial organic acid production [16]. This multi-source organic acid input collectively maximizes the dissolution of recalcitrant calcium-bound P, thereby sustaining plant growth under the severe P limitation characteristic of karst ecosystems [33].

**Figure 12:**
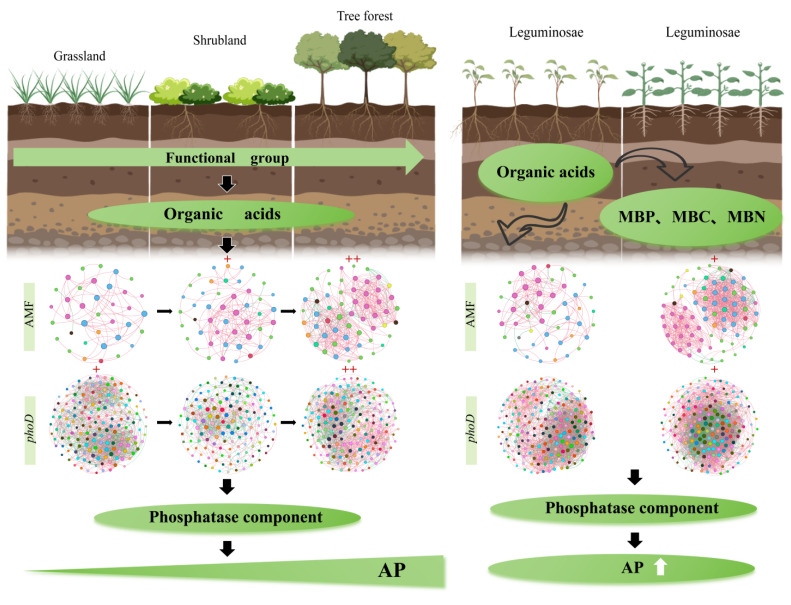
Changes in AP across different functional groups in the karst ecosystem. The red “+” indicates mild microbial association, while "++" indicates a stronger degree of microbial association.

Therefore, this study concludes that the increase in P availability in karst soils is associated with plant–microbe synergistic interactions. Oxalic acid is linked to this process through a dual pathway: statistical association with the dissolution of calcium-bound P, and links to higher phosphatase activity via shifts in *phoD*-harboring bacteria and AMF. The random forest model identified microbial biomass, phosphatase activity, and oxalic acid content as key predictors of available P in the tree stage, Furthermore, Structural Equation Modeling (SEM) suggested that the diversity of *phoD*-harboring bacteria and AMF is significantly associated with available P accumulation via positive links to phosphatase activity and soil P fractions, with this synergistic effect being more pronounced in leguminous plants. These results collectively suggest that organic acids facilitate phosphorus availability by fostering synergistic microbial networks and enzyme activities, a process particularly prominent in leguminous plants, thereby providing empirical alignment with our third hypothesis. In summary, plants alleviate P limitation in karst ecosystems through the exudation of oxalic acid, which increases microbial diversity and ultimately activates the combined mechanism of soil phosphatase activity (Figure 12), providing a theoretical basis for ecological restoration.

## 5. Conclusions

In summary, this study moves beyond qualitative descriptions to provide a quantitative benchmark for how plant functional diversity is linked to phosphorus (P) acquisition in karst ecosystems. Our results demonstrate that the magnitude of oxalic acid exudation is a primary factor associated with soil P availability, with significant variations observed among different plant groups. By quantifying these group-specific physiological responses and microbial synergies, this work provides a theoretical basis for selecting optimal restoration species based on their association with mobilizing soil phosphorus. Under the conditions studied, this process is associated with a dual mechanism: firstly, the linkage between Ca^2+^ chelation and the dissolution of calcium-bound P, which represents the dominant chemical pathway in these high-calcium alkaline environments; secondly, the enrichment of P-solubilizing bacteria carrying the *phoD* gene and the strengthening of the arbuscular mycorrhizal fungi network. These shifts are associated with higher phosphatase activity and microbial biomass P. The synergistic interaction between AMF and *phoD* bacteria is linked to higher P acquisition efficiency, with leguminous plants exhibiting a more significant association. Importantly, as this study is purely observational and no management interventions were conducted, the results from our Structural Equation Modeling (SEM) and statistical analyses identify associations and directional links only, rather than establishing direct cause-and-effect relationships. While these quantitative findings offer a robust framework, it is important to note that this study is primarily based on correlation analysis within specific karst regions. Further controlled experiments are needed to establish causality and elucidate the molecular mechanisms controlling organic acid–microbe interactions. Future research should focus on extending this framework to different karst geological ecosystems to assess its universality and application potential, ultimately alleviating soil P limitation and promoting sustainable restoration in karst areas.

## Figures and Tables

**Table 1:** Information on dominant plants in three vegetation restoration stages.

Stages	Vegetation Description	Species
Grassland	Dominated by *Miscanthus floridulus*, with low vegetation coverage and a thin litter layer.	*Pueraria lobata* (Legume), *Miscanthus floridulus* (Non-legume), *Imperata cylindrica* (Non-legume)
Shrubland	Dominated by *Vitex negundo*, with a moderately developed litter layer and a patchy understory of grasses and lianas.	*Phanera championii* (Legume), *Pterolobium punctatu* (Legume), *Vitex negund* (Non-legume), *Ligustrum sinense* (Non-legume)
Tree forest	Dominated by *Cladrastis platycarpa*, with high vegetation coverage, a thick litter layer, and a diverse understory of grasses and lianas.	*Cladrastis platycarpa* (Legume), *Cipadessa cinerascens* (Non-legume), *Radermachera sinica* (Non-legume), *Machilus nanmu* (Non-legume), *Cornus macrophyll* (Non-legume), *Mallotus philippensis* (Non-legume)

## Data Availability

The raw data supporting the conclusions of this article will be made available by the authors on request.

## Supplementary Materials

The following supporting information can be downloaded at: https://www.mdpi.com/article/10.3390/microorganisms14050952/s1, Figure S1: Changes in relative abundance of arbuscular mycorrhizal AMF and *phoD* at the order level in different functional groups. G, grass; SH, shrub; T, trees; L, leguminous plants; NL, non-leguminous plants. Table S1: Physical and chemical properties of rhizosphere soil in different plant functional groups. Data S1: Organic acid data during the growth season.
