# Supplementary material for: Plant-Derived Organic Acids Are Linked to Arbuscular Mycorrhizal Fungi and phoD-Harboring Bacteria Associated with Improved Soil Phosphorus Availability Across Plant Functional Groups in Karst Ecosystems
Source: Microorganisms. 2026 Apr 23;14(5):952. doi: 10.3390/microorganisms14050952 (PMC13209602; doi:10.3390/microorganisms14050952)
Supplement: Supplementary file 1 [file microorganisms-14-00952-s001.zip › Supplementary File.pdf]

Table S1. Physical and chemical properties of rhizosphere soil in different functional groups plants

Different functional groups plants	SOC g/kg	NO_3^--N mg/kg	NH_4^+-N mg/kg	TN g/kg	MBC mg/kg	MBN mg/kg	pH	$ExCa^{2+}$ mg/kg	$ExMg^{2+}$ mg/kg
Grass	32.37±1.21c	25.33±5.54b	3.36±0.26b	4.92±0.71b	694.65±88.65c	227.18±45.74b	7.76±0.05b	31.76±1.97c	30.37±1.49b
Shrub	53.51±1.52b	43.61±7.88b	5.62±0.34b	5.87±0.25b	1355.28±115.37b	391.77±49.54a	7.88±0.02a	41.30±0.94b	37.50±0.36a
Tree	74.57±1.77a	68.05±6.49a	16.66±1.32a	8.17±0.29a	1931.22±105.75a	481.94±48.56a	7.83±0.01b	48.38±0.96a	38.50±0.36a

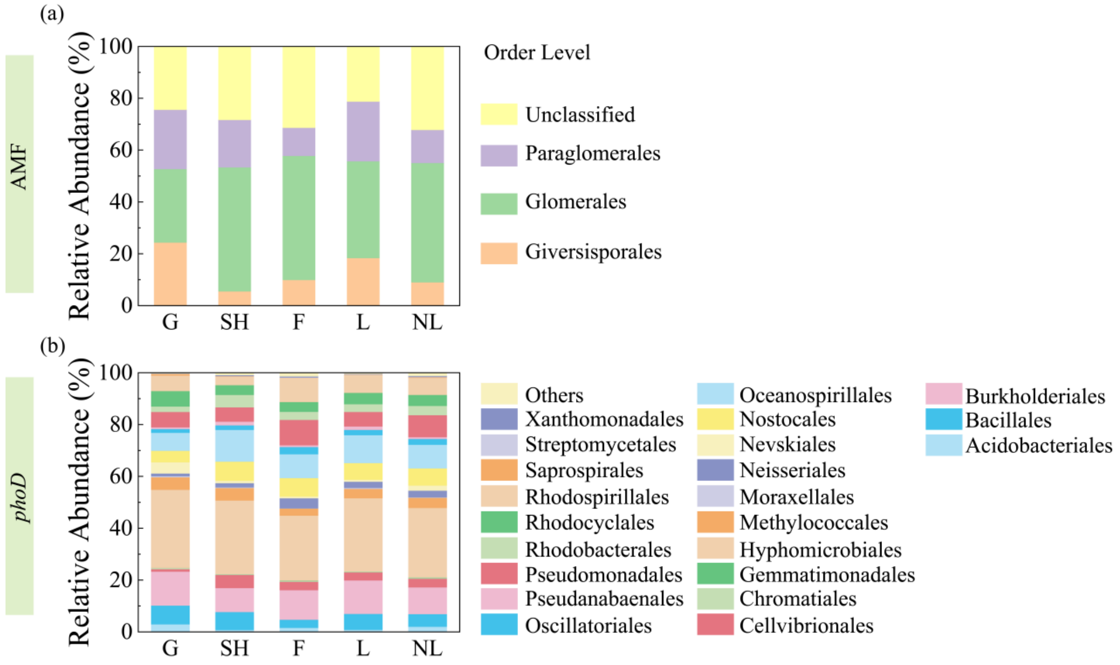

Figure S1. Changes in relative abundance of arbuscular mycorrhizal AMF and *phoD* at the order level in different functional groups. G, grass; SH, shrub; T, trees. L, leguminous plants; NL, non-leguminous plants.
